# A Rare Case of Monteggia Associated With a Terrible Triad: A Case Report

**DOI:** 10.1002/ccr3.71369

**Published:** 2025-10-31

**Authors:** Mehdi Tiotour, Mohamad Sheibani, Salman Azarsina, Mohammadsajad Mirhosseini, Amirhossein Hajialigol

**Affiliations:** ^1^ Department of Orthopedic Surgery Shahid Madani Hospital, Alborz University of Medical Sciences Karaj Iran; ^2^ Alborz Office of Universal Scientific Education and Research Network (USERN), Alborz University of Medical Sciences Karaj Iran

**Keywords:** coronoid process, elbow dislocation, elbow joint, Monteggia fractures, radial head fractures, triad injury

## Abstract

The coexistence of Monteggia fracture and terrible triad injury represents an exceptionally rare and complex elbow trauma. A systematic, staged surgical approach focusing on bony and ligamentous reconstruction, followed by early mobilization, is crucial for restoring joint stability and function.

## 1 Introduction

Elbow injuries involving fracture‐dislocations are among the most complex to manage due to the joint's intricate anatomy and its reliance on both osseous and ligamentous structures for stability [1]. The Monteggia lesion, originally described by Giovanni Battista Monteggia in 1814, typically consists of a proximal ulna fracture with dislocation of the radial head [2]. In contrast, the “terrible triad” of the elbow, first outlined by Hotchkiss in 1996, involves a posterior elbow dislocation, fracture of the radial head, and fracture of the coronoid process [3]. Each injury independently poses significant treatment challenges and is associated with a high risk of complications such as stiffness, heterotopic ossification, and recurrent instability. When these two injuries co‐occur—Monteggia and the terrible triad—the treatment complexity is magnified, yet such cases remain scarcely documented [4].

Recent literature has sought to redefine traditional classifications. Giannicola et al. proposed the concept of “Monteggia‐like” lesions, incorporating more complex elbow fracture‐dislocation patterns that may not fit into Bado's original classification but still demand similarly aggressive surgical intervention. Additionally, the Wrightington classification now emphasizes a three‐column concept to guide the surgical management of complex elbow injuries [5, 6]. Monteggia fractures are typically categorized using the Bado classification, which identifies four distinct types based on the direction of radial head dislocation and the associated ulna fracture pattern [7]. Type I involves a forward (anterior) dislocation of the radial head along with an ulna shaft fracture angulated in the same direction. Type II refers to a backward (posterior) or backward‐sideways (posterolateral) dislocation of the radial head with a corresponding ulna fracture that opens in the same direction. Type III includes a sideways (lateral) dislocation of the radial head and a fracture in the proximal portion of the ulna, often near the metaphysis. Type IV, which applies to our patient, is the least common and involves fractures of both the radius and ulna, accompanied by dislocation of the radial head [8]. This combination usually indicates a high‐impact trauma and presents a significant challenge in achieving stable surgical repair. This case report presents a unique instance of combined Monteggia lesion and terrible triad in a young adult male, illustrating the complexity of managing such hybrid injuries and the importance of a coordinated surgical strategy.

## 2 Case History/Examination

A 35‐year‐old male presented after a 3‐m fall onto his outstretched left arm. Clinical findings included elbow deformity and swelling, without open wounds. Neurovascular status was intact. Initial radiographs revealed a proximal ulna fracture with posterior dislocation of the radial head, a comminuted radial head fracture, and a coronoid process fracture—consistent with a combined Monteggia lesion and terrible triad.

**FIGURE 1:**
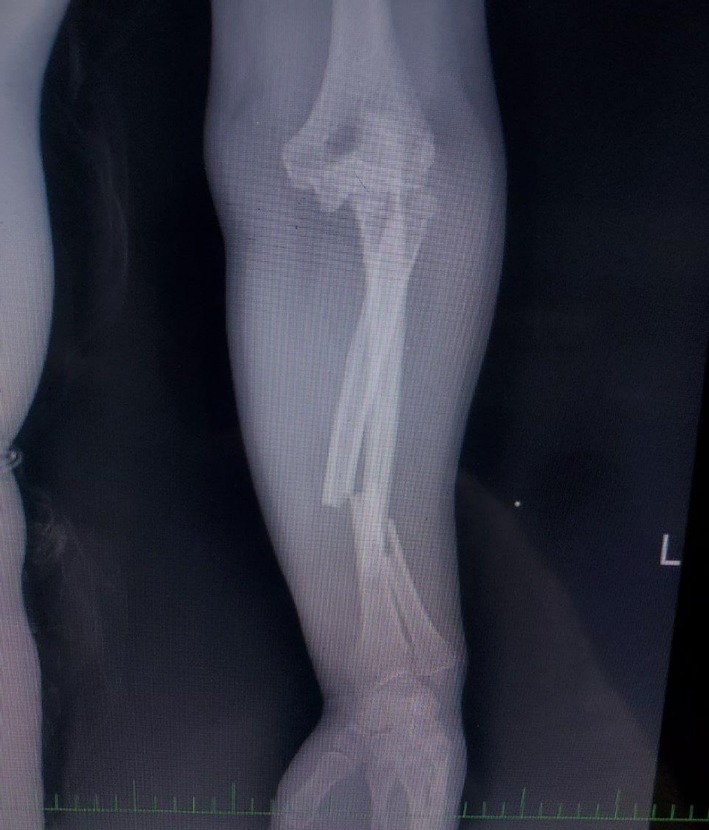
Initial radiograph showing proximal ulna fracture with posterior dislocation of the radial head.

**FIGURE 2:**
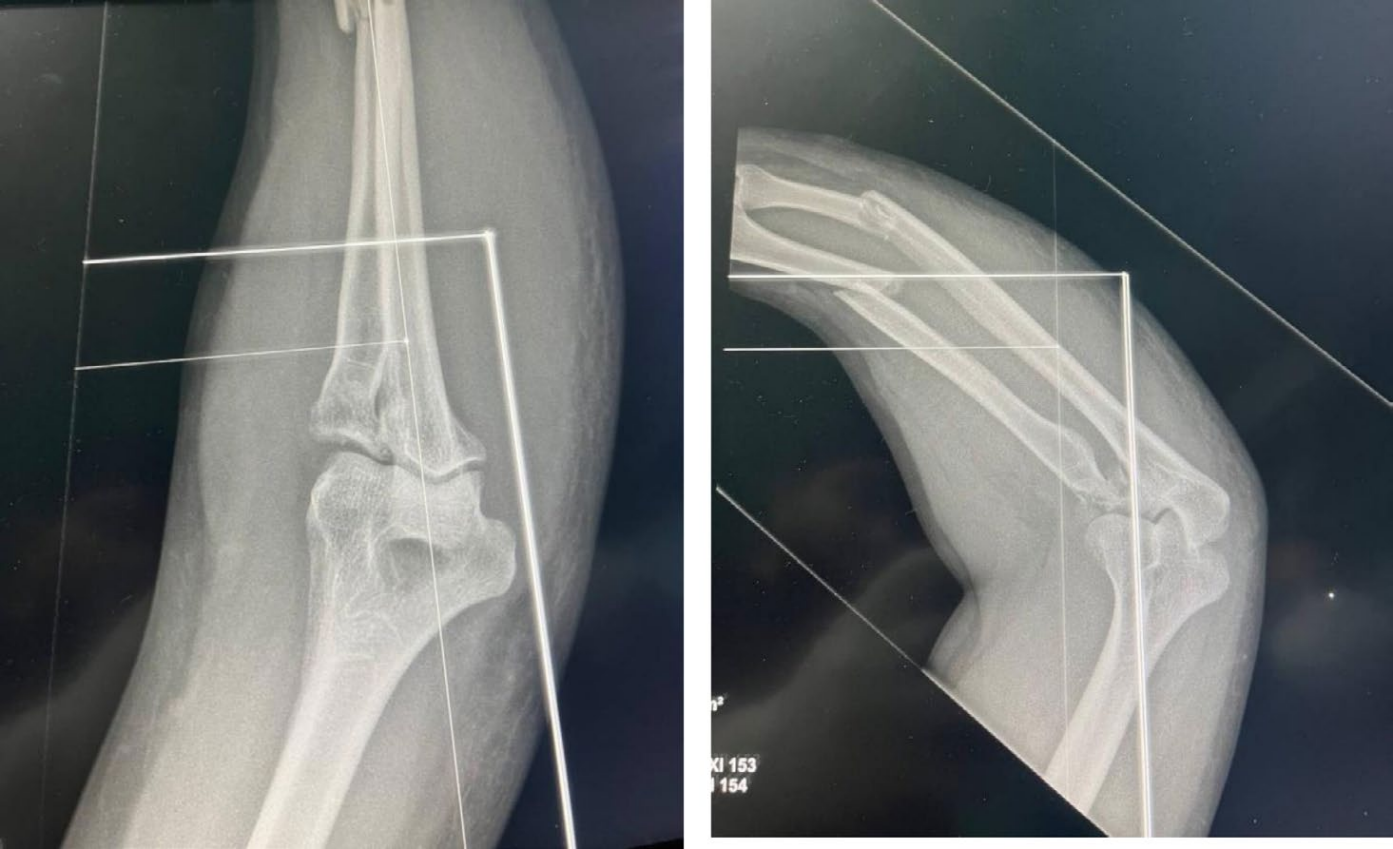
Radiograph after closed reduction of the dislocated elbow performed in the emergency room under sedation.

**FIGURE 3:**
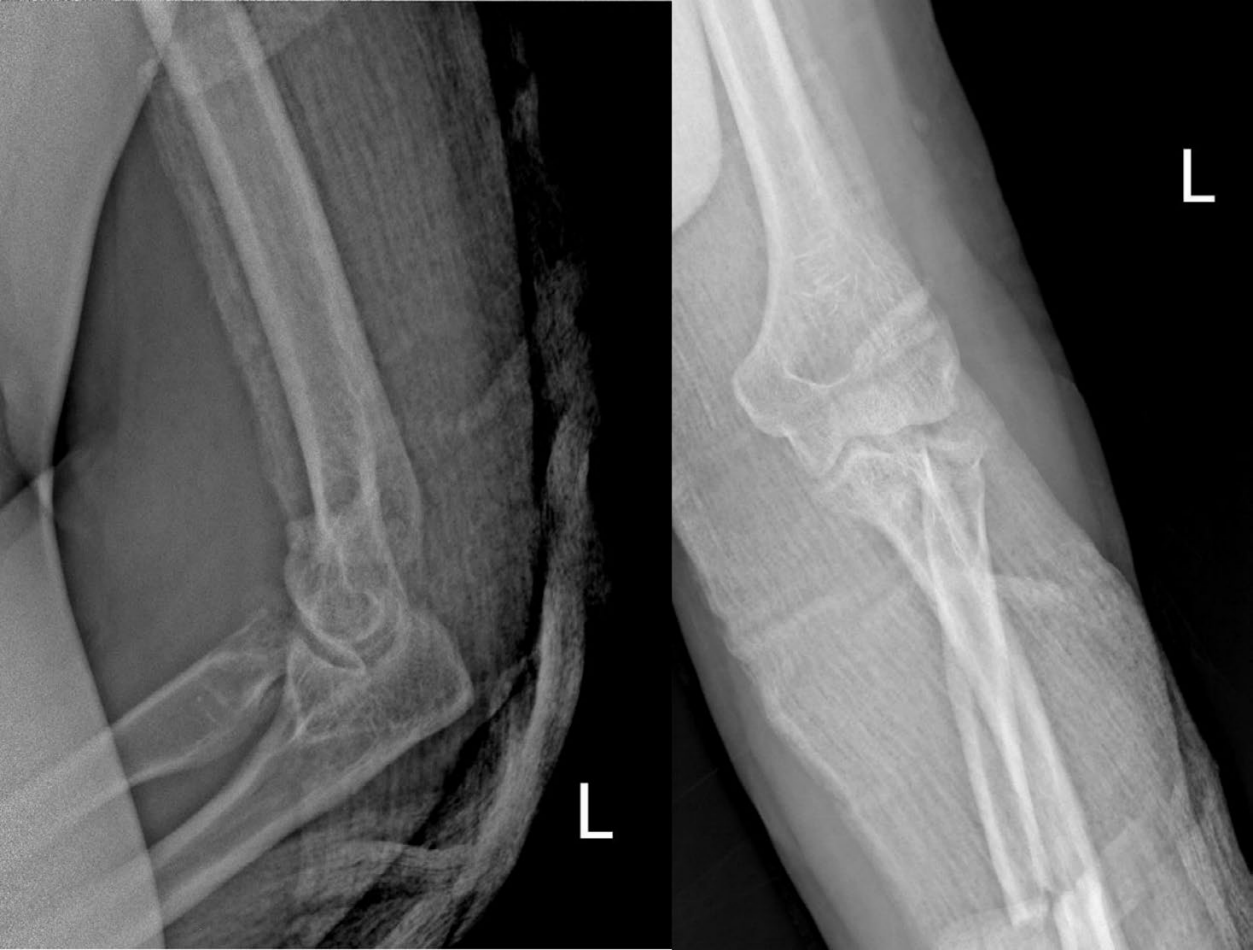
Lateral view of the elbow demonstrating associated coronoid and radial head fractures prior to definitive surgery.

Plain radiographs (Figure 1) revealed a complex injury pattern, including a proximal ulna fracture, posterior dislocation of the radial head, and associated fractures of the radial head and coronoid process—consistent with a combined Monteggia fracture‐dislocation and terrible triad injury of the elbow. The patient was initially managed in the emergency department with closed reduction of the dislocated elbow under sedation and application of a long‐arm posterior splint (Figure 2). Following stabilization, the patient was scheduled for definitive surgical management. Given the complexity of the injury and the need for dual stabilization of both the forearm and elbow, a staged surgical approach was planned (Figure 3). A CT scan of the elbow was obtained and is included as a supplementary video. This confirmed the fracture morphology and alignment, supporting the diagnosis of a combined Monteggia fracture‐dislocation and terrible triad injury.

In the first operative stage, the patient was positioned supine, and fixation of the forearm fractures was carried out. Using the Henry approach, the radius was exposed and fixed with a 3.5 mm locking compression plate (LCP). The ulna was accessed through a separate posterior approach and similarly stabilized with a 3.5 mm LCP. Intraoperative assessment revealed significant elbow instability, prompting the need for a second‐stage procedure. The second surgery was performed with the patient in the lateral decubitus position. A posterior midline incision was used to access the elbow. On exposure, the radial head was found to be severely comminuted and irreparable. Due to the lack of access to a radial head prosthesis, radial head resection was performed. The coronoid fracture was too small for hardware fixation and was treated with capsular plication. The lateral collateral ligament (LCL) complex was repaired using non‐absorbable sutures through bone tunnels via the Kocher interval. Despite these reconstructions, residual elbow instability remained.

**FIGURE 4:**
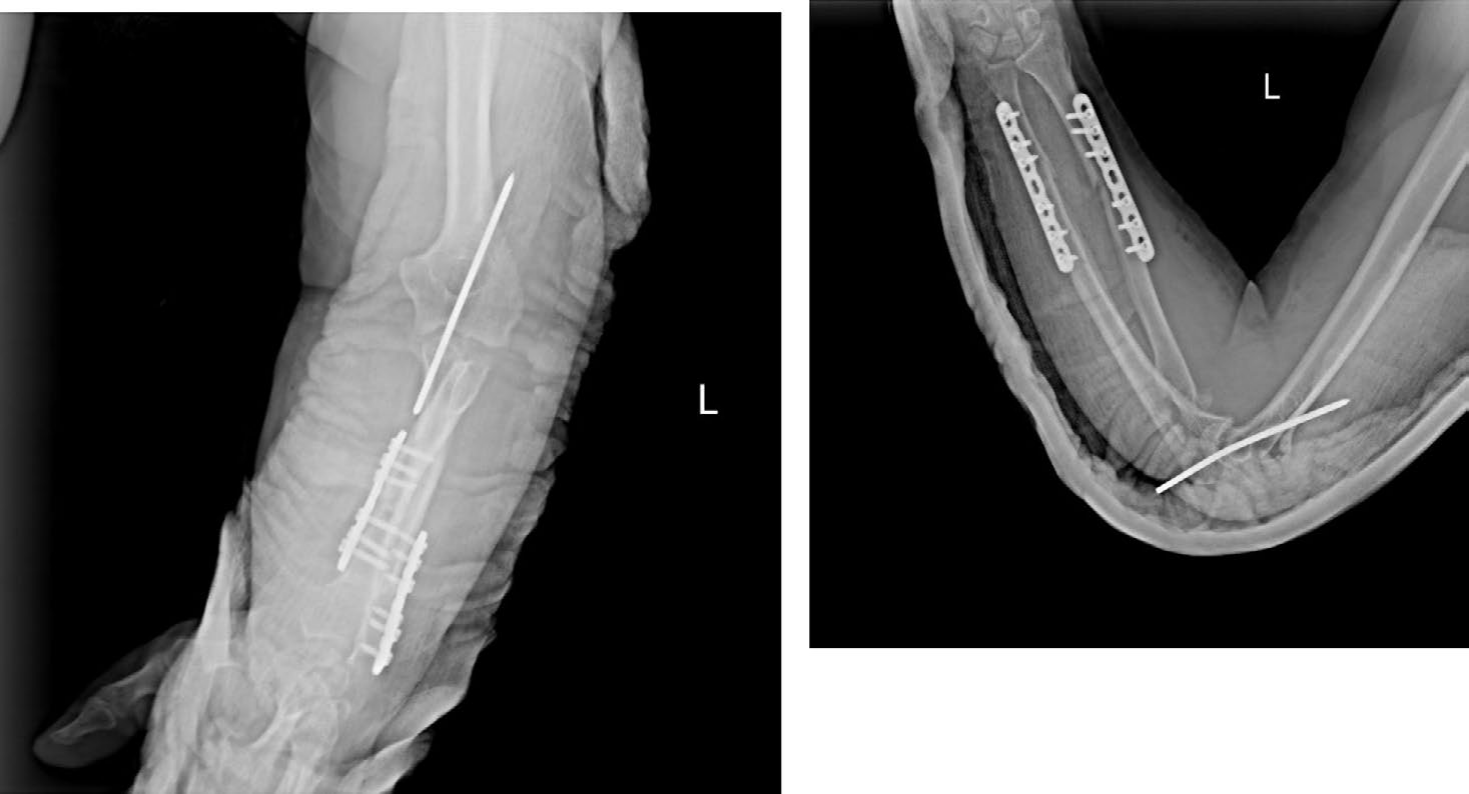
Immediate postoperative radiograph showing restored alignment and internal fixation of the ulna and radius.

To enhance joint stability, a trans‐olecranon Kirschner wire was inserted to temporarily stabilize the elbow in slight flexion. The anterior capsule was also repaired to support anterior stability. The patient tolerated the procedures well and was discharged with intact neurovascular status, immobilized in a posterior splint. Postoperative rehabilitation began within 48 h under close supervision, following a structured protocol for elbow motion and strengthening. Follow‐up radiographs (Figure 4) confirmed appropriate alignment and healing. By 3 months postoperatively, the patient achieved a functional arc of motion (20°–100° flexion) and full forearm rotation without evidence of instability or infection (Video S1).

## 3 Differential Diagnosis, Investigations, and Treatment

The differential diagnosis included isolated Monteggia and terrible triad injuries. However, imaging confirmed the presence of both. Initial management included closed reduction and splinting.

A staged surgical plan was executed:

1. Stage 1: Internal fixation of the radius and ulna using 3.5 mm LCPs via Henry and posterior approaches.
2. Stage 2: Due to irreparable radial head fracture and absence of a prosthesis, radial head resection was performed. Coronoid fracture was addressed with capsular plication; LCL was reconstructed with sutures through bone tunnels. The trans‐olecranon K‐wire was maintained for 4 weeks and subsequently removed prior to initiation of more advanced mobilization.

## 4 Conclusion and Results (Outcome and Follow‐up)

**FIGURE 5:**
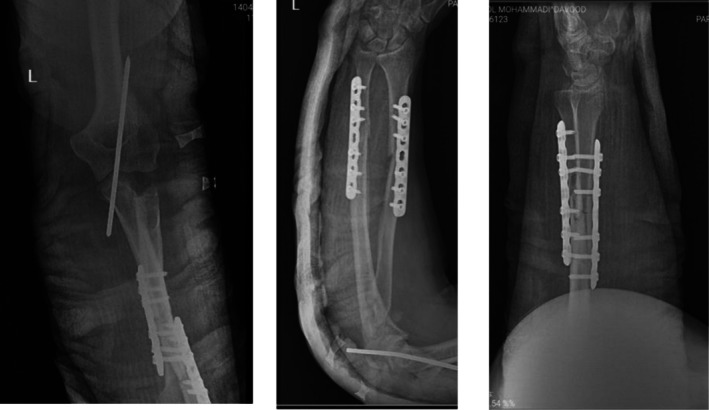
One‐month postoperative radiograph demonstrating maintained alignment and early callus formation.

**FIGURE 6:**
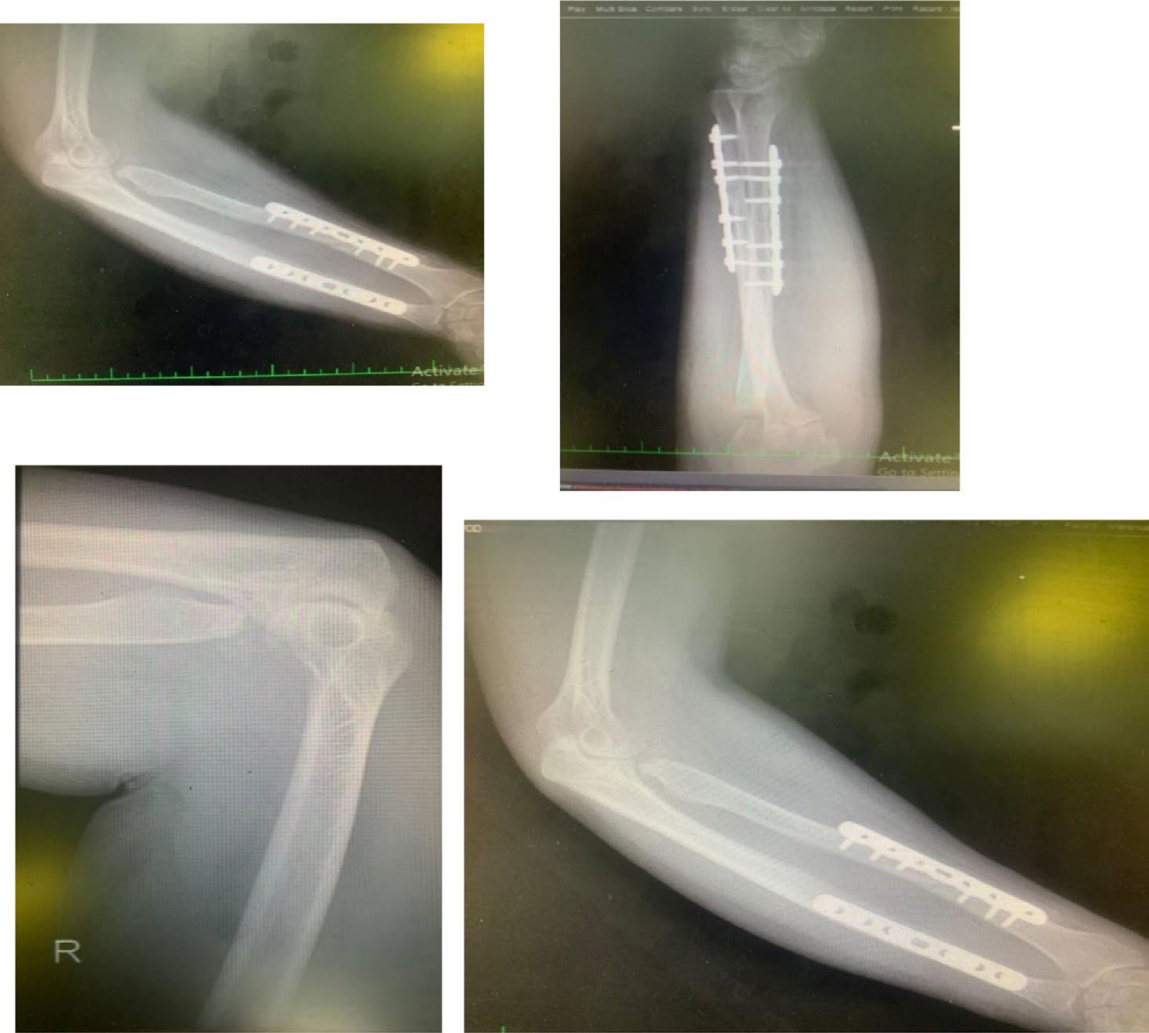
Four‐month postoperative radiograph showing advanced bone consolidation and remodeling.

At the 1‐month follow‐up, radiographs (Figure 5) showed maintained alignment with early callus formation, and the patient had achieved 30°–90° of flexion with partial forearm rotation. By 3 months, functional recovery was notable: the patient regained a motion arc of 20°–100° in flexion, with full pronation and supination, without instability or infection. At the 4‐month follow‐up, radiographs (Figure 6) demonstrated advanced consolidation and remodeling, and the patient had further improvement in function, returning to most daily activities with minimal discomfort. A supplementary video has been provided showing the patient's active elbow movements at 4 months (Video S2).

Despite these encouraging short‐term results, complex elbow fracture‐dislocations are well known for late complications such as stiffness, heterotopic ossification, and post‐traumatic arthritis. Continued long‐term clinical and radiographic follow‐up is planned to monitor for these potential issues and ensure durable functional outcomes.

## 5 Discussion

This case exemplifies the rare and complex scenario of a Monteggia fracture associated with a terrible triad injury, a combination that blurs traditional classification boundaries. Comparing our case to recent reports: Our case echoes several key observations from Matthias Jung et al., who retrospectively reviewed “Monteggia‐like” lesions treated with radial head arthroplasty and reported both functional and radiological outcomes [9]. In that series, radial head replacement was performed in all cases of irreparable radial head fractures, and coronoid and ulnar stabilization were achieved to restore elbow biomechanics. In contrast, our patient underwent radial head resection due to implant unavailability, but we compensated with rigorous soft tissue repair, including LCL and anterior capsule reconstructions, followed by temporary trans‐olecranon stabilization [10]. Both approaches resulted in restoration of elbow stability and early mobilization, with our patient achieving a functional motion arc by 3 months—similar to the arthroplasty group in Matthias Jung et al. However, unlike the arthroplasty cohort, we lack long‐term prosthetic support, which may influence durability. Our case therefore highlights a viable surgical alternative when prosthetic resources are limited, while reinforcing the core premise of Matthias Jung et al.—that biomechanical restoration of both bony and ligamentous structures is essential for optimal short‐term function.

In contrast, Ben Brahim et al. reported a 43‐year‐old male with a diaphyseal ulnar fracture, Mason III radial head fracture, and coronoid fracture, treated with radial head arthroplasty and coronoid fixation. Their outcome was favorable, with early rehabilitation and a structured surgical protocol based on the three‐column concept. Our case similarly followed a comprehensive surgical approach and benefited from early mobilization, resulting in a good functional outcome [11]. These comparisons highlight the importance of early, rigid fixation and ligamentous repair. In line with current protocols, we prioritized bony stability, followed by lateral ligament reconstruction, to ensure a congruent, stable elbow joint—consistent with findings in recent systematic reviews [11].

Another key takeaway from this case is the real‐world challenge of limited resources—in this instance, the unavailability of a radial head prosthesis. While prosthetic replacement is typically favored in cases of severe comminution, we opted for resection due to practical constraints. This highlights the need for adaptable surgical strategies that can still yield satisfactory outcomes when ideal implants or tools are not accessible. The patient's favorable recovery despite this limitation suggests that in selected cases, radial head resection combined with robust ligamentous repair and temporary stabilization may be an acceptable alternative when prosthetic replacement is not feasible.

Moreover, the utility of frameworks such as Giannicola's Monteggia‐like spectrum and the Wrightington classification can aid clinicians in planning treatment for these atypical but severe injury patterns.

## Author Contributions

**Mehdi Tiotour:** conceptualization, data curation, investigation, writing – original draft. **Mohamad Sheibani:** conceptualization, investigation, supervision, writing – review and editing. **Salman Azarsina:** conceptualization, investigation, project administration, supervision, writing – review and editing. **Mohammadsajad Mirhosseini:** data curation, project administration, supervision, writing – review and editing. **Amirhossein Hajialigol:** conceptualization, investigation, software, writing – original draft.

## Ethics Statement

The study was approved by the ethics committee of Alborz University of Medical Sciences and written informed consent was obtained from all participants or their parents before enrolling to the study.

## Consent

The authors have nothing to report.

## Conflicts of Interest

The authors declare no conflicts of interest.

## Supporting information


**Video S1:** Preoperative CT scan (sagittal view) demonstrating proximal ulna fracture with posterior dislocation of the radial head and associated coronoid process fracture.

**Video S2:** Four‐month postoperative clinical evaluation showing the patient's functional elbow motion with full forearm rotation and stable joint alignment.

## Data Availability

Supporting Information is available in the online version.
